# Involvement of extracellular vesicles in the macrophage-tumor cell communication in head and neck squamous cell carcinoma

**DOI:** 10.1371/journal.pone.0224710

**Published:** 2019-11-07

**Authors:** Àngela Maria Bellmunt, Laura López-Puerto, Juan Lorente, Daniel Closa

**Affiliations:** 1 Otolaryngology Department, Hospital Vall d’Hebron, Universitat Autònoma de Barcelona (UAB), Barcelona, Spain; 2 Department of Experimental Pathology, Institut d’Investigacions Biomèdiques de Barcelona, Consejo Superior de Investigaciones Científicas (IIBB-CSIC), Institut d’Investigacions Biomèdiques August Pi i Sunyer (IDIBAPS), Barcelona, Spain; College of Medicine, National Cheng Kung University, Tainan Taiwan, TAIWAN

## Abstract

**Background:**

Exosomes are cell-derived vesicles that mediate cellular communication in health and multiple diseases, including cancer. However, its role in head and neck cancer has been poorly defined. Here, we investigated the relevance of exosomes in the signaling between larynx cancer cells and macrophages.

**Methods:**

Exosomes from THP1 macrophages and BICR18 cells (a larynx squamous cell carcinoma cell line) were purified and their role in the cancer cell migration, macrophage phenotype and immunosuppressive activity was evaluated. The activation of STAT3 signal transduction in macrophages in response to exosomes obtained from cancer cells was also evaluated.

**Results:**

Macrophages foster the cancer cell migration and this effect is mediated by exosome signaling. On the other hand, exosomes also induce the expression of IL-10 in macrophages and PD-L1 in cancer cells, thus resulting in the promotion of an immunosuppressive environment. Moreover, we observed that the effects induced in cancer cells are mediated by the exosome-depending activation of STAT-3 signal transduction pathway.

**Conclusions:**

Our study indicates that exosomes released by both macrophages and cancer cells plays a critical role in tumor progression in larynx cancer and might be a potential target for therapeutic intervention in head and neck cancer.

## Background

Head and neck cancer is the 6th most common cancer worldwide and over 833,000 new patients worldwide are diagnosed each year [1,2]. Laryngeal carcinoma still causes a relevant mortality, being squamous cell carcinoma (SCC) the most prevalent histology [3]. It has being strongly related to tobacco exposure and to alcohol intake while other factors, as human papillomaviruses, plays a minor and uncertain causal role [4,5]. Despite recent improvements in the therapeutic strategies, treatment failures still occur and the development of new therapeutic strategies as well as an increased understanding of the biomarkers involved in the process are required. Recently, first line treatments in recurrent or metastatic head and neck squamous cell carcinoma with anti-PD1 agents have shown a survival improvement over standard therapy [6].

In the progression of cancer, tumor microenvironment is composed either for cancer cells, extracellular matrix and a variety of non-cancer cells, including inflammatory cells, fibroblasts and endothelial cells [7,8]. Communication cell-to-cell is of utmost importance for tumor growth and progression and relevant differences have been observed in treatment response and patient survival depending on the immune cell infiltration in the tumors and matrix [9,10]. Immune cell infiltrate includes tumor-associated macrophages (TAM) that produce a variety of angiogenic, immunosuppressive and growth-related factors, thus contributing to the malignancy of the tumor [11]. Macrophages display marked phenotypic heterogeneity that can be divided into M1, characterized by the secretion of proinflammatory cytokines, and M2 that contribute to the production of the extra-cellular matrix and encourage tumor progression. In the initial stages of tumor development, TAM display an M1 phenotype, while in the later stage of neoplastic progression they become polarized toward M2 protumoral phenotype [12].

Immunosuppression is also induced through the overexpression of programmed cell death ligand 1 (PD-L1), a functional ligand of programmed cell death receptor 1 (PD‑1). Binding of tumor cell PD‑L1 to immune T-cell PD‑1 induces the inhibitions of T-cell activation and results in the evasion of antitumor immunity [13]. It has been reported that the presence of macrophages is associated with tumoral PD-L1 expression [14] and macrophages itself could also express PD-L1 [15].

The interplay between cancer and the immune microenvironment is known to be mediated by soluble molecular mediators. However, a fairly recent mechanism based on extracellular vesicles has been described to intervene in cell-to-cell communication. [16]. Extracellular vesicles (EVs), including exosomes and microvesicles, are nano-sized membrane vesicles containing proteins and nucleic acids that act as intercellular messengers. Initially considered as merely cellular waste product, it is now clear that they play an important role as mediators of intercellular communication in many physiological and pathological processes, particularly in inflammation and cancer [17,18]. These vesicles have been reported to be involved in macrophage polarization or in cell migration in different cancer models [19]. The purpose of this work is to characterize the potential involvement of extracellular vesicles in the macrophages—cancer cells dialogue in an *in vitro* model of larynx squamous cell carcinoma.

## Materials and methods

### Cells

Human THP1 cells (a human leukemia monocytic cell line that can be differentiated into macrophages) were cultured in suspension in RPMI 1640 medium supplemented with 10% fetal bovine serum (FBS; GibcoTM, Thermo Fisher Scientific, Waltham, MA), 2 mM L-glutamine, 100 U/ml penicillin and 100 μg/ml streptomycin. Cells were differentiated to macrophages through a first incubation with 100 nM phorbol 12-myristate 13-acetate (PMA) (Sigma-Aldrich, St. Louis, MO) for 48 h. After that, the PMA-containing media was discarded and replaced with fresh media without PMA for a further 24 h.

Adherent keratinocyte cell line BICR18, derived from a metastasic lymph node of a laryngeal squamous cell carcinoma (SCC), were cultured in DMEM medium, supplemented with 10% FBS, 2 mM L-glutamine, 100 U/ml penicillin and 100 μg/ml streptomycin. The experiments were performed when 70% of confluence was achieved.

In some experiments, BICR18 cells were cultured at the bottom of the well plate and co-cultured with macrophage-differentiated THP1 cells physically separated using a transwell system of 24-well plate, 0.4 μm pore filter, (Corning, VWR International Ltd., UK) over 24 h.

All cells were grown in a humidified atmosphere of 95% air, 5% CO2 at 37°C.

### Exosomes isolation

In order to generate exosome-free medium, exosomes present in fetal bovine were removed by an overnight centrifugation at 100 000 g followed by filtration through 0.2-μm syringe-fitted filters (Millipore, Burlington, MA). This exosome-depleted fetal bovine serum was used for cell culture (DMEM supplemented with 10% exome-free fetal bovine serum). For the exosomes’ isolation, cell supernatants were collected and centrifuged at 2 000 xg and 10 000 xg for 10 and 30 min, respectively, at 4°C. The last supernatant was filtered through a 0.22 μm syringe filter (Millipore Burlington, MA) and ultracentrifuged at 120 000 xg for 70 min. After that, the pelleted vesicles were washed with phosphate-buffered saline (PBS) and centrifuged again at 120 000 xg for 70 min [20]. Quality of exosomes preparations was verified by nanoparticle tracking analysis, electron microscopy analysis of their size and shape and thus by determining the presence/absence of exosomal markers TSG101, and ALIX by Western Blot. The amount of exosomes obtained was also quantified by measuring their protein content using a Bradford assay, as previously described [20].

### Electron microscopy

For imaging, isolated exosomes were fixed in 2% paraformaldehyde, adsorbed in formvar-coated nickel grids for 20 min and negative stained with 4% uranyl oxalate. Grids were air dried and observed in a JEOL-1010 Transmission Electron Microscope (Jeol USA, Peabody, MA) at 80 kV.

### Nanoparticle tracking analysis

The size distribution and concentration of exosomes were measured using a NanoSight LM10 machine (NanoSight, Salisbury, UK). All the parameters of the analysis were set at the same values for all samples and three 1 min-long videos were recorded in all cases. Background was measured by testing filtered PBS, which revealed no signal.

### SDS-PAGE and Western blot

Exosomal protein was extracted in RIPA Buffer in the presence of protease inhibitors, separated by a 12% SDS-PAGE and transferred to a PVDF membrane (Immun-Blot, Bio Rad, Hercules, CA). Membranes were blocked for 1 h in 5% nonfat milk in PBS, followed by overnight incubation with antibodies against Alix and TSG101 as well as Calnexin as negative control (Acris antibodies, Herford, Germany). Blots were washed and incubated for 1 h 30 min at room temperature with a DyLight 800-conjugated secondary antibody. Immunoreactive bands were visualized with an Odyssey Infrared Imaging System (LI-COR Biosciences, Lincoln, NE).

### Exosomes and cells staining

For internalization assays, exosomes were labeled with the PKH26 red fluorescent cell linker dye (Sigma-Aldrich, St. Louis, MO) for 5 min. The staining reaction was stopped with 3% BSA for 1 min. In order to remove the unbound dye, exosomes were washed three times with PBS using 300 KDa Nanosep centrifugal devices (Pall Corporation, New York, NY). Cells were labeled with PKH67 green fluorescent cell linker dye, following the same protocol.

### Exosomes uptake

To monitor exosomes uptake, 3 μg/ml (3,9 x 10^7^ part/ml) of labeled BICR18-Exos were added to THP-1 macrophages and incubated for 2 h. The same experiment was performed with BICR18 cells, which were incubated with 3 μg/ml (2,5x10^7^ part/ml) of labeled THP1-exos for 2 h. The concentration of Exosomes was selected according to previous in vitro studies [21]. Exosomes internalization was analyzed by fluorescence microscopy imaging.

### Migration assay

BICR-18 cells were grown until confluence in 12 well plates and treated with 0,5 μg/ml of mitomycin C (Roche, Basel, Switzerland) for 2 h to arrest proliferation. Then, the scratch wound was made on the monolayer of cells using a 200 mL pipette tip followed by a wash with PBS. At this point, co-culture with THP1-cells using a transwell system was started or, alternatively, 5 μg/ml of THP1-Exos were added to the corresponding wells. After 24 h, the migration of cells was captured using a phase contrast microscope (Leica Microsystems, Wetzlar, Germany), the wound area was measured, and cell migration quantified using Cell^R software. In additional experiments, increasing concentrations of the exosome secretion inhibitor GW4869 (Sigma-Aldrich, St. Louis, MO) was added to the cell culture. In this experiment, the same amount of DMSO was added to all the culture wells.

### RT-PCR and qPCR

Total RNA was extracted by phenol-chloroform extraction and ethanol precipitation using TRizol^®^ reagent (Invitrogen, Carlsbat, CA). Isolated RNA was diluted in RNAse-free water and stored at -80°C. RNA samples were quantified using a Nanodrop ND-1000 device. A reverse transcription reaction was performed on 1 μg RNA sample using iScript reagents (Bio Rad, Hercules, CA) and following the manufacturer’s specifications. The mixture was incubated at 25°C for 5 min, 42°C for 30 min, and 85°C for 5 min. Finally, it was diluted in RNAse-free water so that the final concentration was 10 μg/ml and stored at -80°C.

Subsequent qPCR amplification was performed using iTaq^®^ SYBR Green Supermix (Bio Rad, Hercules, CA) and the corresponding primers: IL1β Forward: 5’-GGACAAGCTGAGGAAGATGC-3’ Reverse: 5’-TCGTTATCCCATGTGTCGAA-3’; MRC1 Forward: 5’-GGATGGATGGCTCTGGTG-3’ Reverse: 5’-TCTGGTAGGAAACGCTGGT-3’; IL-10 Forward: 5’-GTT CTT TGG GGA GCC AAC AG -3’ Reverse: 5’-GCTCCCTGGTTTCTCTTCCT-3’; PD-L1 Forward: 5’-TATGGTGGTGCCGACTACAA-3’ Reverse: 5’- TGACTGGATCCACAACCAAA-3’; SOCS-3 Forward 5’-CCACCTGAGTCTCCAGCTTC-3’ Reverse: 5’-GTTCAGCATTCCCGAAGTGT-3’; GAPDH Forward: 5’-GATCATGAGCAATGCCTCCT-3’ Reverse: 5’-TGTGGTCATGAGTCGTTCCA-3’. Reactions were performed in duplicate and threshold cycle values were normalized to GAPDH gene expression. The specificity of the products was determined by melting curve analysis. The ratio of the relative expression of target genes to GAPDH was calculated by using the ΔC(t) formula.

### Immunofluorescence

To monitor STAT3 translocation, BICR18 cells were incubated on coverslips and treated with THP1-Exos for 30 min. Following treatment, cells were fixed with 3.5% formaldehyde for 5 min, permeated with 0,1% triton X-100 and blocked with serum. The staining was performed by incubating with anti-STAT3 antibody (Santa Cruz Biotechnology, Santa Cruz, CA) and Alexa fluor 488-conjugated anti-goat secondary antibody (Molecular Probes, Eugene, OR) previous to mounting with aqueous mounting medium. Nuclear localization was examined by fluorescence microscopy. In some wells, STAT-3 inhibitor -5,15-Diphenyl-21H,23H-porphine (5,15-DPP) (Sigma-Aldrich, St. Louis, MO) was added.

### Statistics

Statistical analysis was performed with Graphpad Prism software. Data are presented as mean ± SEM. Differences between groups were analysed using a two-tailed Student’s t-test for comparison of two groups and by One-way analysis of variance (ANOVA) followed by Tukey’s post-test when comparing three or more groups. Statistical significance was considered when p<0.05.

## Results

### Characterization of exosomes from BICR18 and THP1 cells

**Fig 1:**
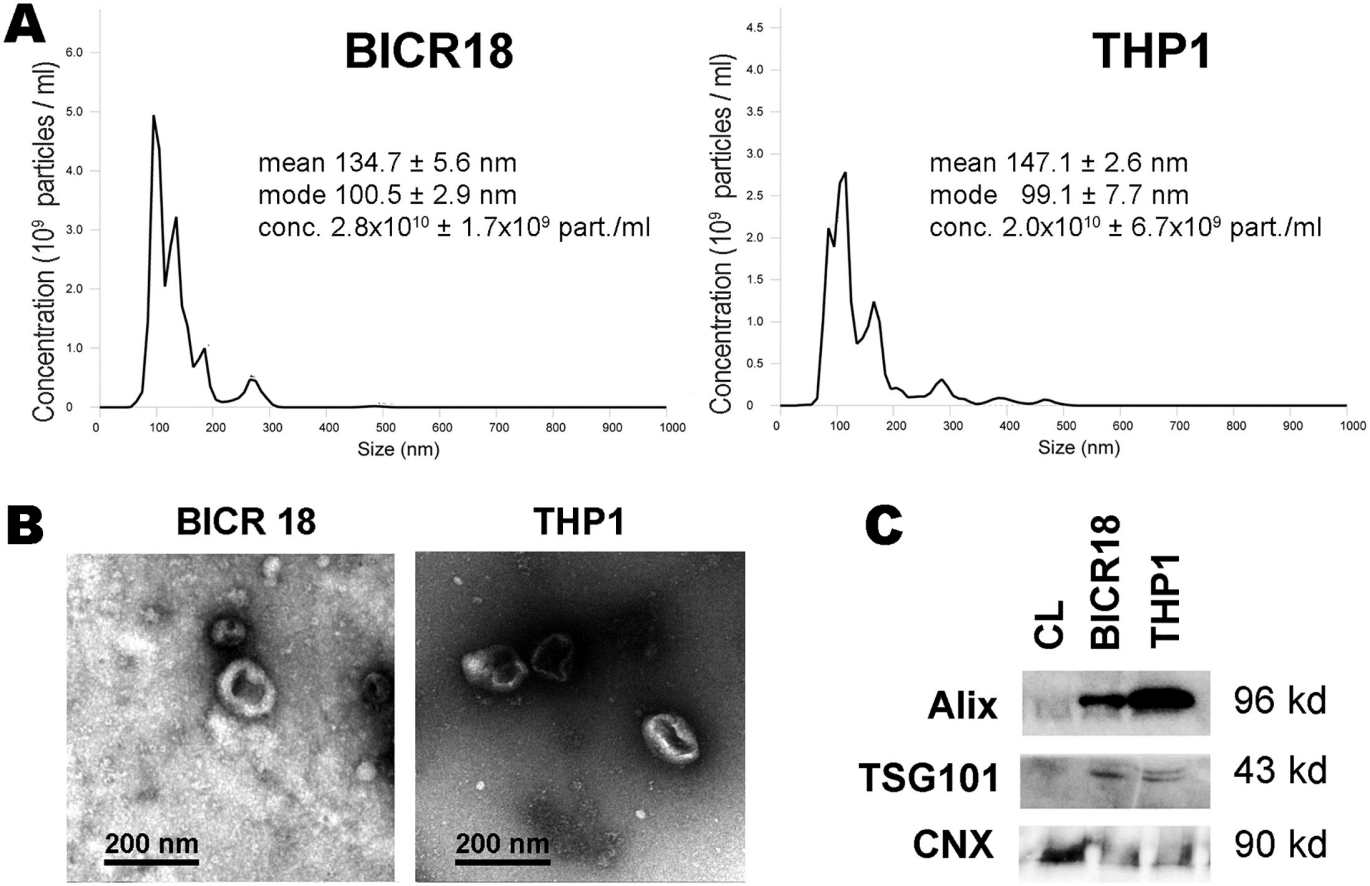
Characterization of extracellular vesicles. **A**: Size distribution, evaluated by nanosight, indicates that sizes are compatible with exosomes. **B**: Electron microscopy revealed the “donut-like” appearance characteristic of exosomes. **C**: Western blot of exosomes samples and cell lysates (CL) to confirm the presence of classical exosome markers (ALIX, TSG101) and the absence of endoplasmic reticulum contamination (Calnexin CXN).

Size Distribution evaluated by nanosight indicates that the average size of purified exosomes from BICR 18 cells ranges between 82 and 179 nm (average of mode size 99 nm) and between 74 and 200 nm (average of mode size 111 nm) for THP-1 cells purified exosomes (Fig 1A). Nanovesicles released by BICR-18 and THP1 cells were analyzed by electron microscopy and images showed vesicles with a size and appearance compatible with exosomes (Fig 1B). Finally, Western blot analysis confirmed the presence of the exosomes-specific markers TSG101 and Alix, sustaining that these vesicles were compatible with exosomes. In addition, the absence of Calnexin, an endoplasmic reticulum marker, confirmed that the potential contamination of exosomes populations with vesicles from other cellular compartments was minimal. (Fig 1C).

### Extracellular vesicle uptake

**Fig 2:**
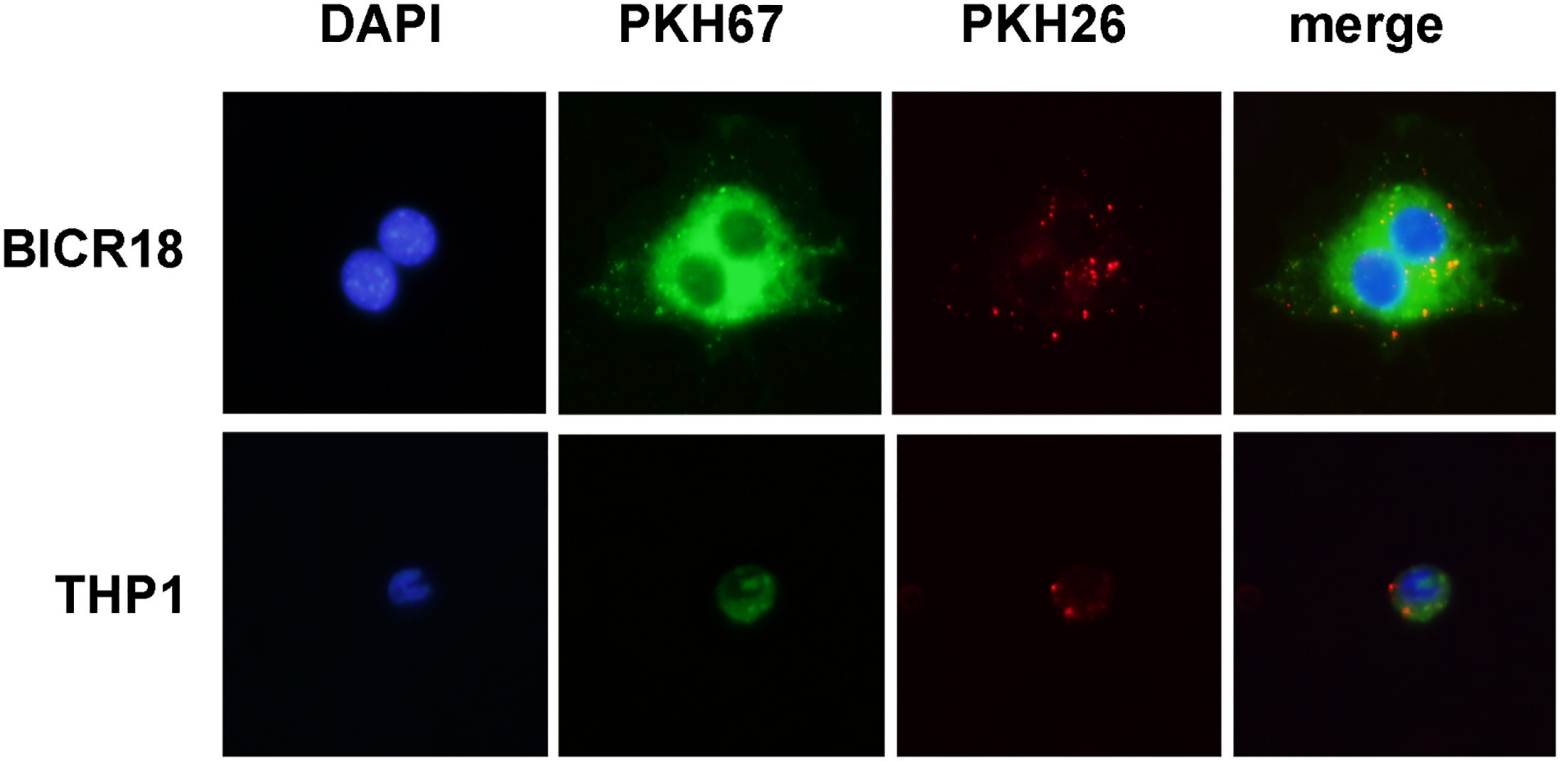
To determine the exosome uptake, BICR18 cells (above) stained with PKH67 (green) were incubated 2 h with 3 μg/ml of PKH26 stained exosomes (red) obtained from THP1 cells. In parallel experiments, THP1 cells (below) were incubated with exosomes obtained from BICR18 cells. In both cases, exosome uptake can be observed.

By fluorescence microscopy we observed that both macrophages and BICR18 could uptake PKH26-stained exosomes. Fig 2 shows THP1 uptake of BICR18-derived exosomes (below) and BICR18 uptake of THP1-derived exosomes (above). In both cases, cells were stained with PKH67 (green) while exosomes with PKH26 (red). Nuclei were stained with DAPI (blue).

### Macrophages promote BICR18 cell migration through exosome mediated signals

**Fig 3:**
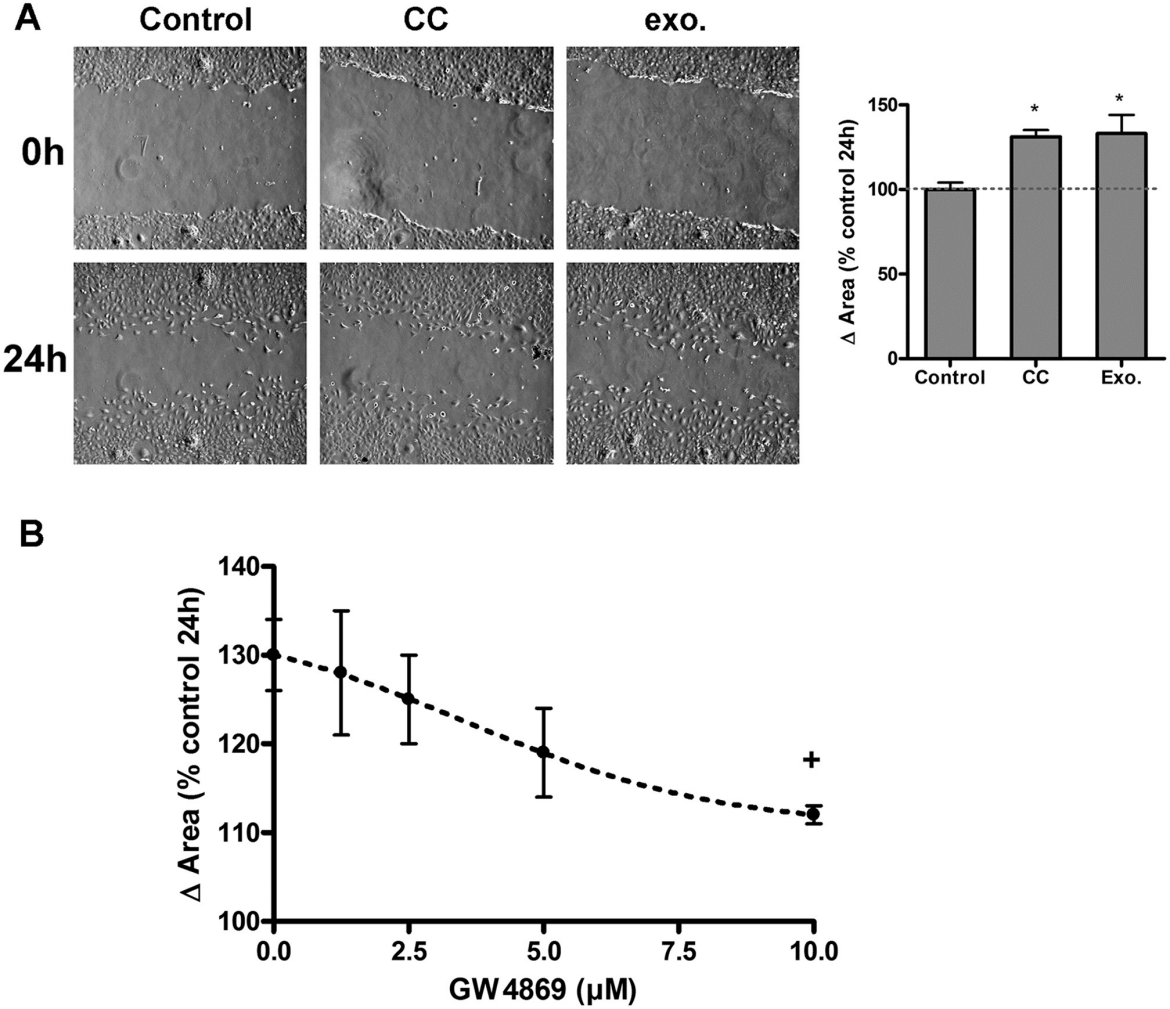
A) Microscopic images showing the BICR18 migratory pattern in control, co-cultured (CC) with THP1 macrophages or treated with 3 μg/ml THP1-exosomes. Both Co-culture and exosomes increases the cellular migration of BICR18 cells. B) The inhibition of exosome secretion with GW4869 prevented the increase of migration induced by coculture. * = p < 0.05 vs Control + p<0.05 vs untreated.

Migration assays were performed to determine the effect of macrophages on BICR18 cell migration ability. The scratch assay is displayed in Fig 3A; a straight wound area was generated in each culture well at the beginning of co-culture. The presence of macrophages resulted in a significantly increased process of wound closure compared with the control cells. A similar increase was obtained when, instead of co-culture, cells were incubated with 15 ng/ml of exosomes purified from THP-1 cells.

The involvement of exosomes was also evaluated by treating cells with increasing concentrations of the exosome secretion inhibitor GW4869 (Fig 3B). The increase observed in the process of wound closure observed in the co-culture was abolished by exosome secretion inhibition.

### Tumor cells modifies macrophage phenotype

When activated, macrophages phenotypes could be classified in three different groups. The first group is the classically activated macrophages, or M1 macrophages, that act as immune effector cells secreting inflammatory cytoquines as IL-1B. A second group is the wound-healing macrophages or M2a macrophages that promote tissue remodeling and are characterized by the expression of mediators as Mannose Receptor (MRC1). Finally, the third group is the regulatory macrophages or M2b macrophages that limit inflammation by generating antiinflammatory mediators as IL-10. Although this classification could be useful, is important to remember that it only represents some points in the broad spectrum of phenotypes acquired by macrophages [20].

**Fig 4:**
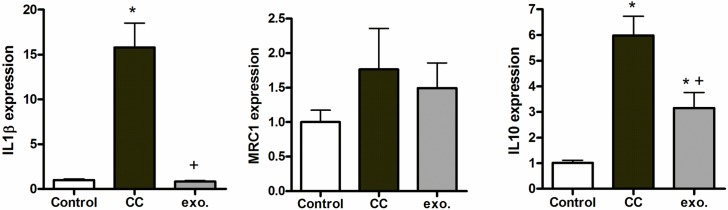
mRNA expression of M1 (IL1β) and M2 (MRC1 and IL-10) markers in THP1 macrophages cocultured 24 h with BICR18 cells (c.c.) or treated with 3 μg/ml exosomes (Exo.) obtained from BICR18 cells. * = p < 0.05 vs Control; + = p < 0.05 vs c.c.

Coculture of BICR18 cells with THP1-derived macrophages resulted in an initial switch to a phenotype characterized by an increased generation of both IL1β and IL10 (Fig 4). Interestingly, treatment with BICR18-derived vesicles only resulted in the induction of IL-10 while no changes were observed on IL1B and MRC1.

### PD-L1 expression on macrophages and BICR18 cells is modulated through exosome mediate signals

**Fig 5:**
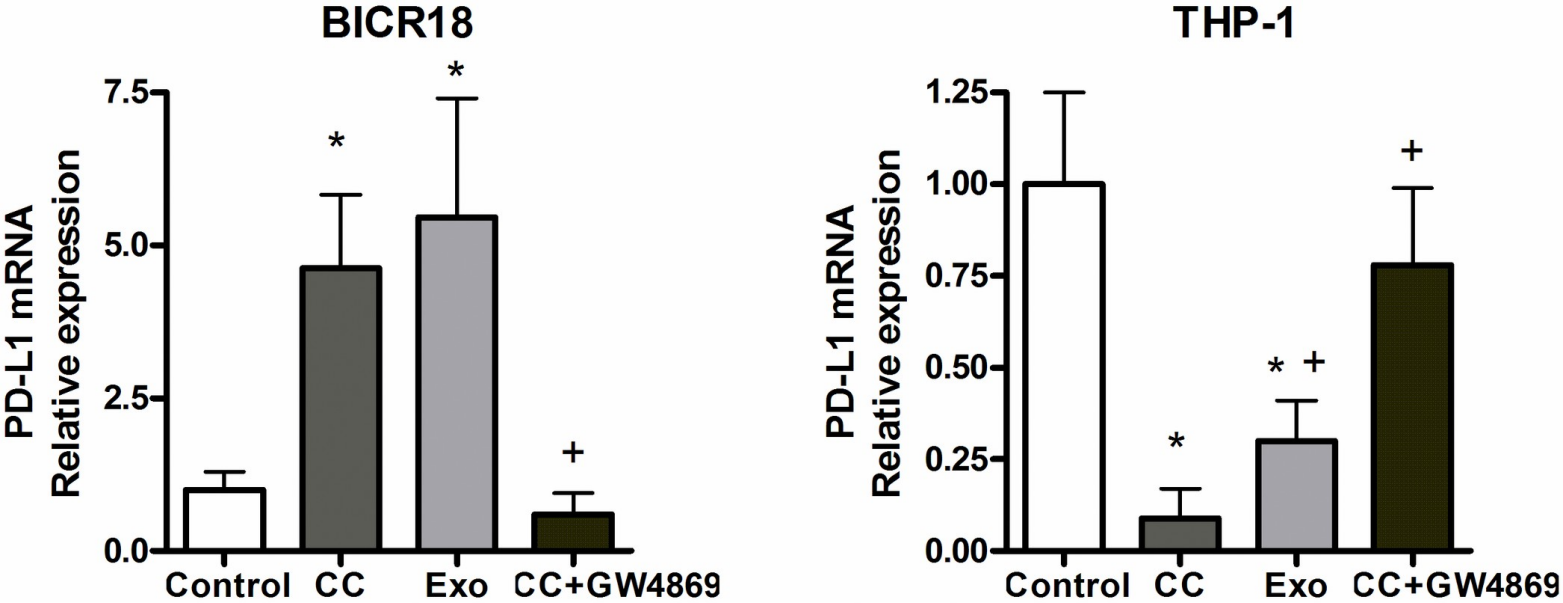
mRNA expression of PD-L1 in BICR18 or THP1 cells after 24 h co-culture or treatment with 3 μg/ml of exosomes obtained from the corresponding cells. * = p < 0.05 vs Control; + = p < 0.05 vs c.c.

Changes in the expression of PDL1 when THP1 macrophages and BICR-18 cells were co-cultured revealed an opposite pattern. In tumor cells, the expression of PD-L1 was induced while in macrophages PD-L1 was inhibited. The same pattern was observed when instead of co-culture, cells were incubated with the corresponding exosomes (Fig 5). Inhibition of exosome secretion with GW4869 treatment abolishes the changes observed in co-culture.

### Induction of PD-L1 in BICR18 cells by exosomes is mediated by STAT3 activation

**Fig 6:**
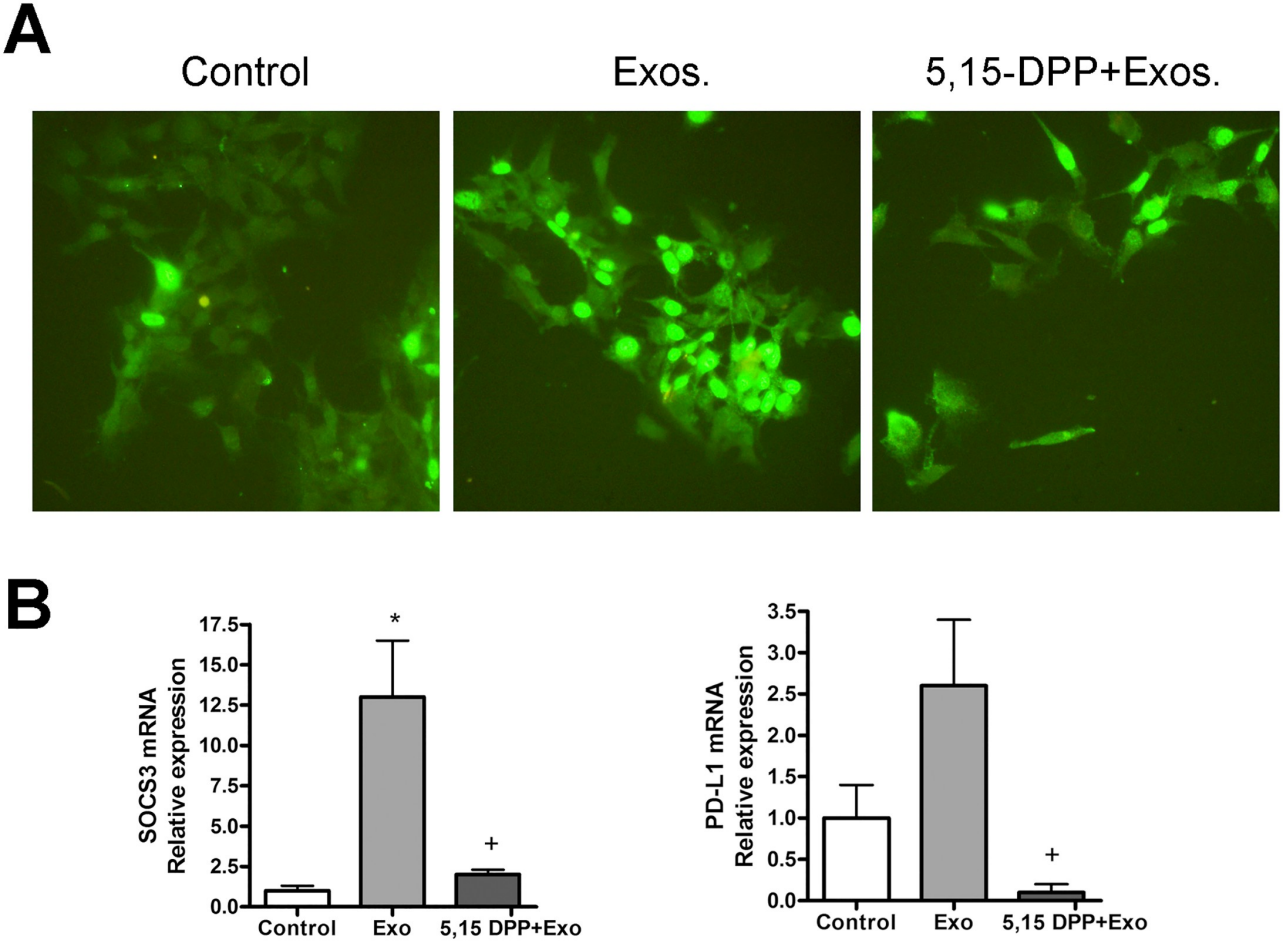
**(A)** Immunofluorescent analysis of STAT3 subcellular location in BICR18 cells. In control cells STAT3 was found mainly in the cytoplasm, although also it was also detected in the nuclei. Treatment with exosomes obtained from THP1 macrophages promoted the activation and almost total nuclear translocation of STAT3. This activations was prevented by treatment with STAT3 inhibitor 5,15-DPP. **(B)** STAT3 inhibition with 5,15-DPP results in complete inhibition of exosome-induced SOCS3. For PD-L1 the inhibition is even more marked. * = p < 0.05 vs Control; + = p < 0.05 vs Exo.

To evaluate the potential involvement of STAT3 in the exosome mediated signaling, we assessed by fluorescence microscopy the nuclear localization of this transcription factor. Treatment of BICR18 cells with THP1-derived exosomes promoted the nuclear translocation of STAT3 (Fig 6A). This effect was inhibited when 5,15-DPP was added to the culture medium. In addition, the expression of SOCS3, a STAT3-induced JAK inhibitor, was evaluated. We found that exosomes triggered the expression of STAT-3 and the blockage of STAT3 activation also resulted in the inhibition of SOCS3. Finally, the PD-L1 expression induced in BICR18 cells by THP1- derived exosomes was also inhibited by 5,15-DPP treatment (Fig 6B).

## Discussion

The interaction between macrophages and tumor cells plays a central role in the progression of cancer and in the induction of mechanisms to evade the anti-tumor response. The paracrine loops between TAMs and cancer cells triggered during cancer progression are mediated by a number of soluble mediators but in the recent years it has become more evident that the involvement of exchange of exosomes among these cells also plays a relevant role in these processes.

It is well known that macrophages can promote tumor progression. In co-culture experiments we have seen that the presence of macrophages promotes an increase in the migration of the BICR18 cells. It is noteworthy that the same effect can be observed if, instead of co-culturing the cells, we treat the tumor cells with the exosomes generated by the macrophages (Fig 3), indicating that the signals that induce cancer cell migration are incorporated into exosomes. Interestingly, exosomes were obtained from unstimulated macrophages, suggesting that stimulation with cancer cells are not required to induce this effect. These results were confirmed by the inhibition of exosome release treating the cells with GW4869. The increase in migration induced by co-culture was reduced in a dose-dependent manner under GW4869 treatment. Similar results have been reported in pancreatic cancer [21], prostate cancer [22] or hepatocarcinoma [23].

By contrast, exosomes released by BICR18 cells seem to play a limited role in macrophages’ phenotypic changes. Co-culture of macrophages and cancer cells results in an initial switch to a phenotype characterized by high expression of IL-1β and IL-10, while an MRC1 expression remains unmodified (Fig 4). This profile does not match with the classics M1 or M2 phenotypes. However, macrophages showed great plasticity and it has been reported that they shift the initial M1-phenotype to a protumorigenic M2-subtype during tumor progression [24]. On the other hand, exosomes treatment does not induce IL1β nor modifies the expression of MRC1 and the only effect generated by these extracellular vesicles is the induction of IL-10, although in levels lower than that obtained with co-culture. Remarkably, the only cytokine induced by BICR18-exosomes plays an immunosuppressive role [25].

The other immunosuppressive mechanism evaluated is the expression of PD-L1 on both cancer cells and macrophages. PD-L1 expressed on the surface of tumor cells could bind PD-1 receptors on the surfaces of activated T cells, resulting in T cell inactivation [13]. For this reason, strategies to block the PD-1/PD-L1 pathway have been developed for cancer immunotherapy in order to promote T cell functions [26]. Expression of PD-L1 is not restricted to cancer cells and it has been reported that other cell populations, as macrophages, could also express PD-L1 in certain types of cancer [27,28].

We observed that co-culture of cancer cells and macrophages results in the expression of PD-L1 in BICR18 cells and a counterpart relevant inhibition in the THP1 macrophages. Interestingly, both results were similarly observed when cells were treated with the exosomes of opposite cells, instead of co-cultured (Fig 5). In addition, these changes were prevented by treating the cells with GW4869, an inhibitor of exosome secretion. Although these results must be interpreted with caution, they indicate that exosomes could play a relevant role in the regulation of PD-L1 inside the tumor.

Different reports indicate that STAT3 is required for PD-L1 up-regulation in prostate cancer or osteosarcoma cell lines [29,30]. These statements raise the question of the involvement of this transcription factor in the exosome induced PD-L1 up-regulation in BICR18 cell line. First, we assessed whether THP1-derived exosomes resulted in STAT 3 activation in BICR-18 cells. Immunofluorescence assay revealed that, effectively, exosomes treatment is able to trigger the nuclear translocation of STAT-3, and that this effect can be blocked by the STAT3 inhibitor 5,15-DPP (Fig 6A). This activation is in line with previous reports from Ham et al. indicating that breast cancer-derived exosomes carries gp130, a subunit of the IL6 receptor that has the capability to activate JAK tyrosine kinases and the transcriptional factor STAT3 [31]. Next, we evaluated the effect of STAT3 inhibitor on the exosome-induced PD-L1 expression (Fig 6B). We also evaluated the induction of SOCS3, a cytokine-inducible negative regulator of cytokine signaling that is induced by STAT-3. We observed that, alongside with SOCS3, the increase of PD-L1 expression induced by exosomes was inhibited when cells were treated with 5,15-DPP, indicating that exosomes induce PD-L1 expression in BICR18 cells through STAT3 transcriptional pathway.

It is increasingly evident that exosomes play a determining role in signaling between the different cells of the tumor micro-environment. Obviously, it remains to explore whether these *in vitro* experiments reproduce *in vivo*. In the same direction, clinical trials investigating the use of new immunomodulatory drugs like CSF-1 or CSF-1R inhibitors are ongoing, aiming to revert the adverse macrophage phenotype induced by exosomes. Explorations of strategies to inhibit precise exosome uptake or biogenesis are eminently justified.

### Conclusions

In summary, our results indicate that exosomes can promote tumor growth by increasing the migratory capacity of laryngeal cancer cells. These extracellular vesicles also boost an immunosuppressive state by inducing mediators such as IL-10 in macrophages or by eliciting the expression of PD-L1 in tumor cells. Altogether, this points to exosomes as a potential therapeutic target in order to modulate the pro-tumoral characteristics of tumor microenvironment.

## Supporting information

S1 Table Nanoparticle tracking analysis, Scratch assay and PCR data. (XLSX)

S1 Fig Western blot for Alix, TSG101 and CNX. (TIF)

S2 Fig Transmission Electron Microscope images for exosomes released by THP1 cells. (TIF)

S3 Fig Transmission Electron Microscope images for exosomes released by BICR18 cells. (TIF)

S4 Fig SOCS3 Immunofluorescence for control BICR18 cells. (TIF)

S5 Fig SOCS3 Immunofluorescence for exosome-treated BICR18 cells. (TIF)

S6 Fig SOCS3 Immunofluorescence for 5,15-DPP pre-treated and exosome-treated BICR18 cells. (TIF)

## Abbreviations

EVs: Extracellular vesicles
PD-L1: programmed cell death ligand 1
PD-1: programmed cell death receptor 1
PMA: phorbol 12-myristate 13-acetate
SCC: squamous cell carcinoma
STAT3: Signal transducer and activator of transcription 3
SOCS3: Suppressor of cytokine signaling 3
TAM: tumor-associated macrophage
