# Supplementary figures and images for: Involvement of extracellular vesicles in the macrophage-tumor cell communication in head and neck squamous cell carcinoma
Source: PLoS One. 2019 Nov 7;14(11):e0224710. doi: 10.1371/journal.pone.0224710 (PMC6837305; doi:10.1371/journal.pone.0224710)

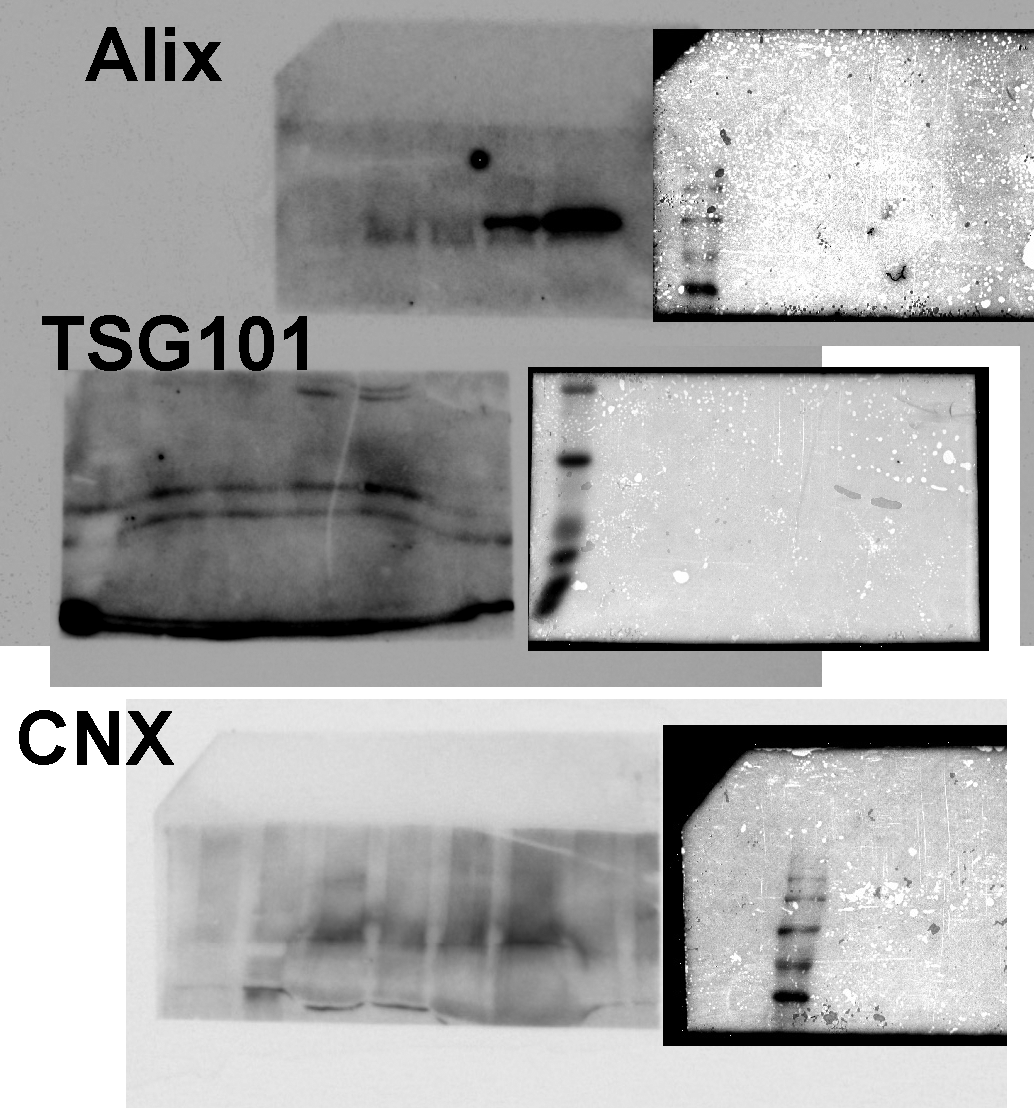

Supplement: S1 Fig — (TIF) [file pone.0224710.s002.tif]

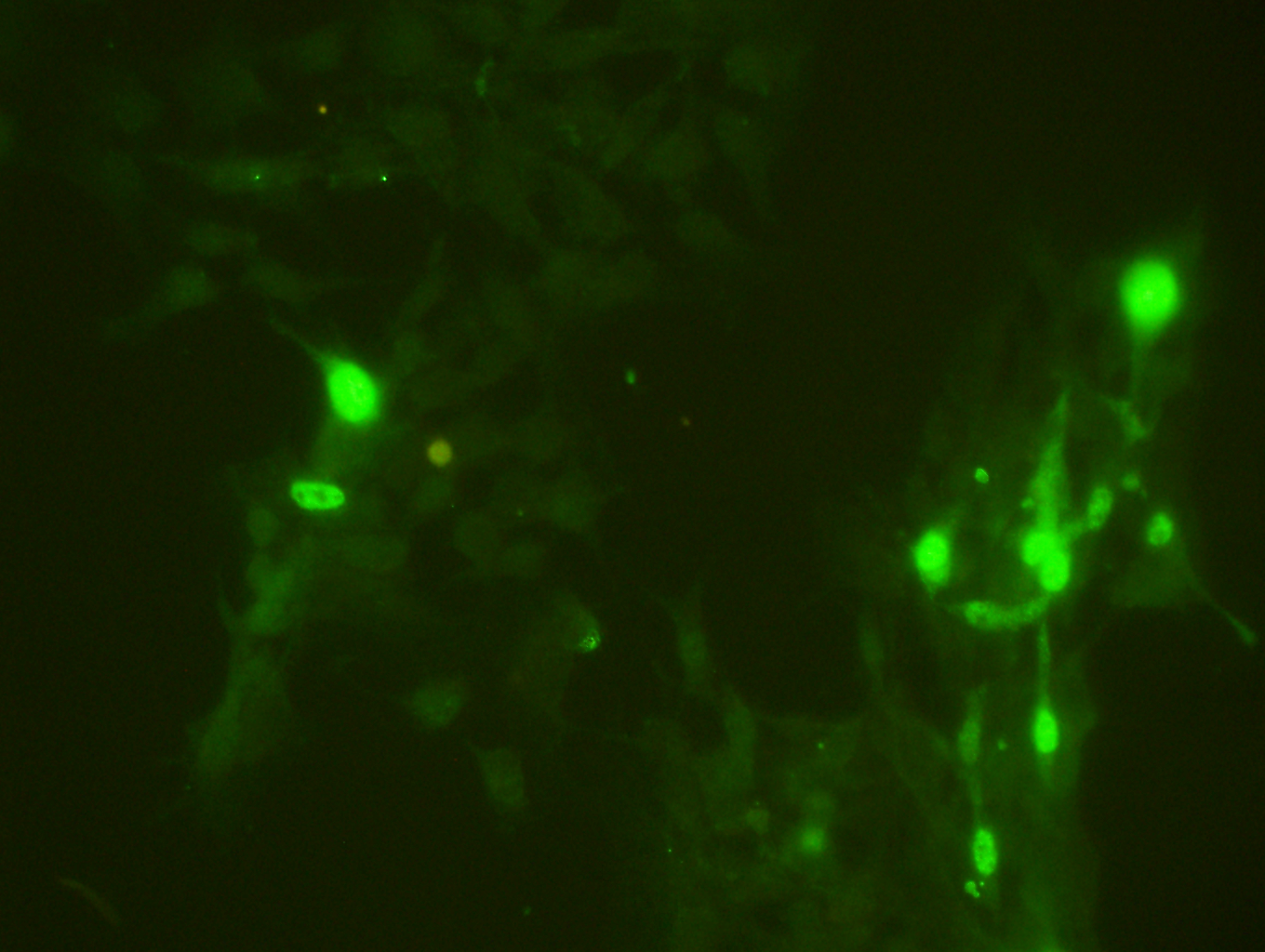

Supplement: S4 Fig — (TIF) [file pone.0224710.s005.tif]

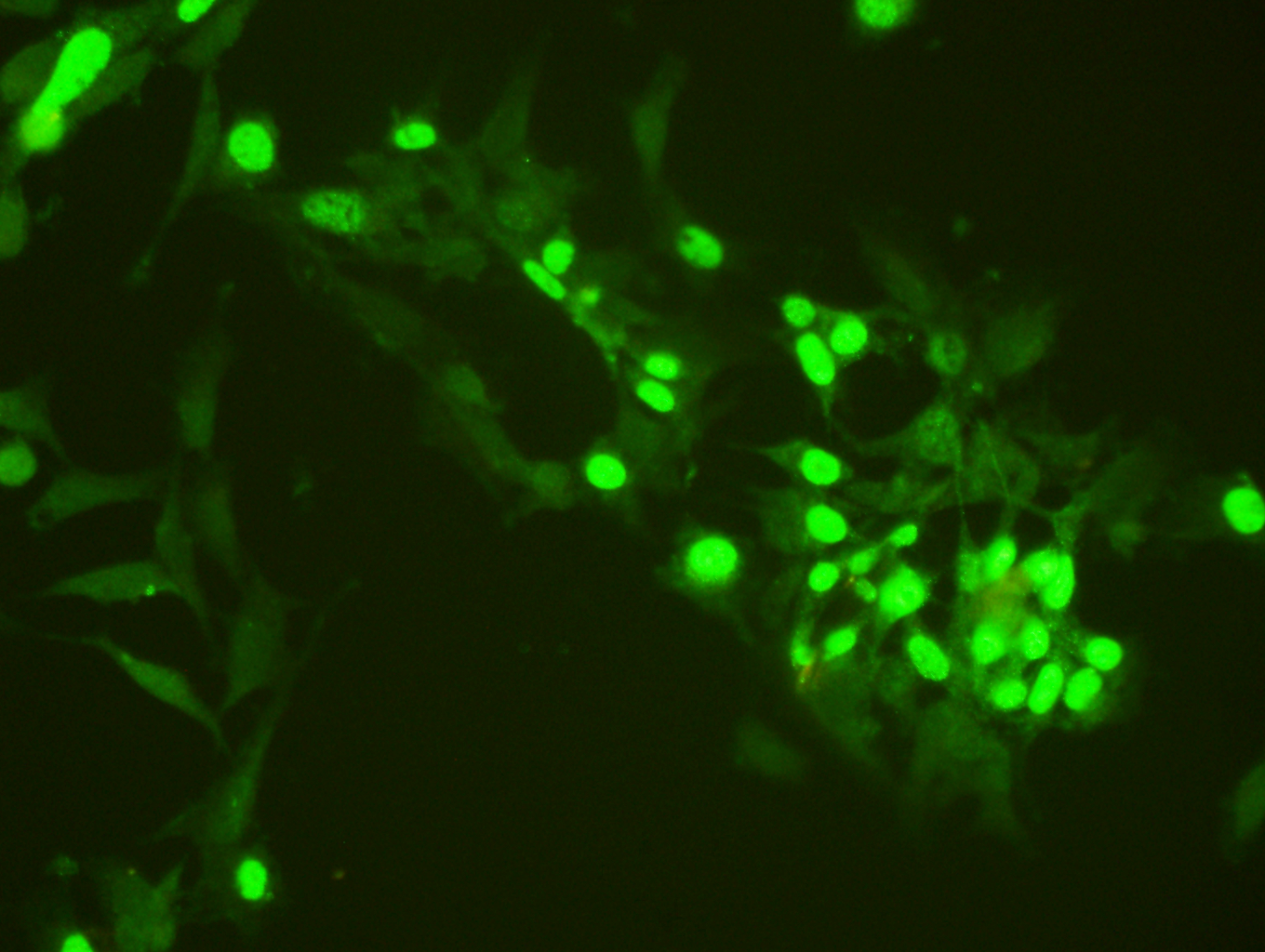

Supplement: S5 Fig — (TIF) [file pone.0224710.s006.tif]

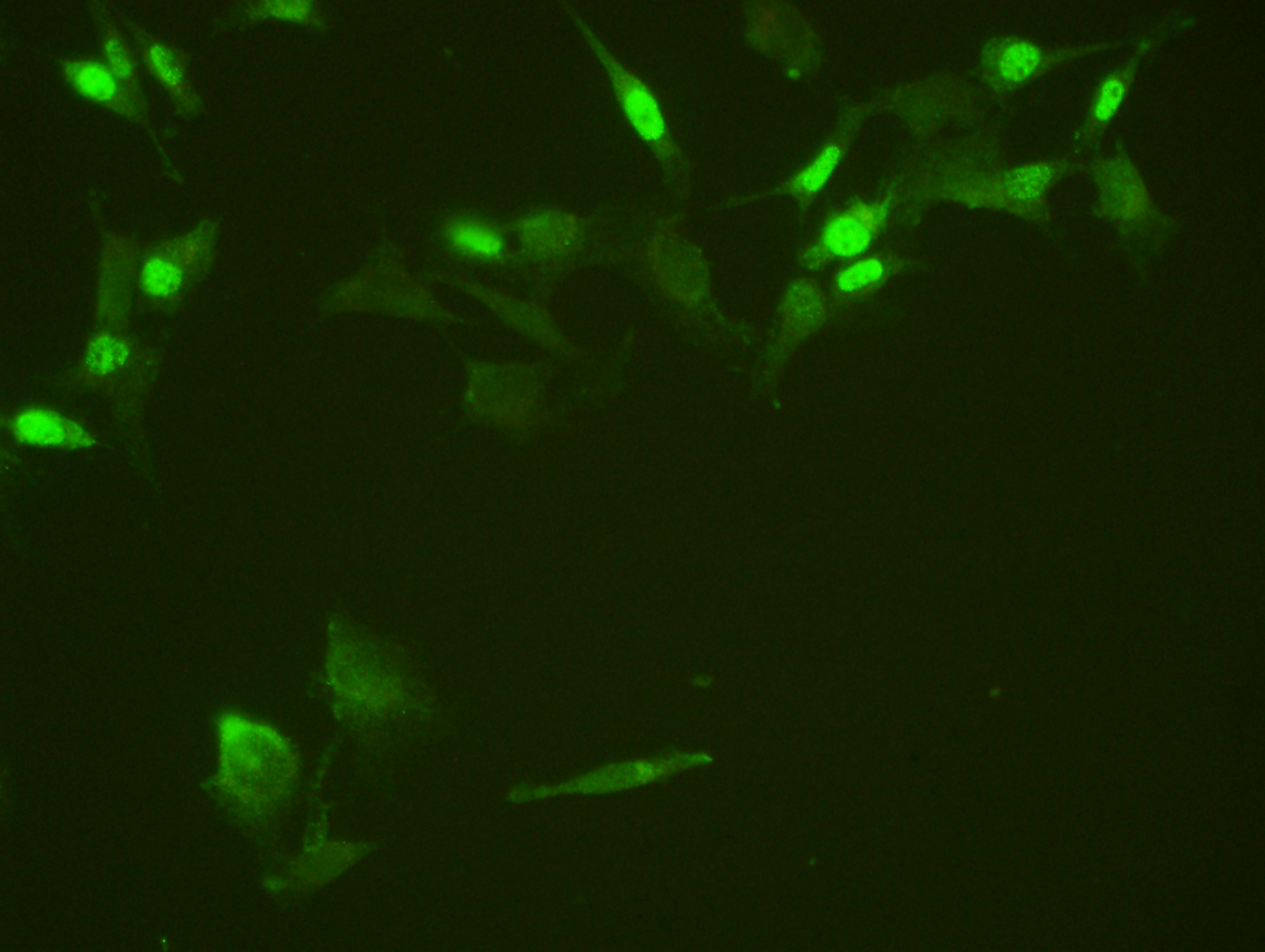

Supplement: S6 Fig — (TIF) [file pone.0224710.s007.tif]
